# Air pollution, neighbourhood walkability and cerebrovascular small vessel disease in patients with cardiovascular disorders along the heart-brain axis

**DOI:** 10.1016/j.cccb.2026.100565

**Published:** 2026-10-03

**Authors:** Ina Rissanen, Erik J. Timmermans, Jeroen de Bresser, Esther E. Bron, Anna E. Leeuwis, Majon Muller, Matthias J.P. van Osch, Betty M. Tijms, Geert Jan Biessels, Ilonca Vaartjes, on behalf of the Heart-Brain Connection Consortium

**Affiliations:** a Julius Center for Health Sciences and Primary Care, University Medical Center Utrecht, Utrecht University, Utrecht, the Netherlands; b Department of Radiology, Leiden University Medical Center, Leiden, the Netherlands; c Department of Radiology & Nuclear Medicine, Erasmus MC, Rotterdam, the Netherlands; d Alzheimer Center Amsterdam, Neurology, Vrije Universiteit Amsterdam, Amsterdam UMC location VUmc, Amsterdam, the Netherlands; e Department of Medical Psychology, Amsterdam UMC, Vrije Universiteit Amsterdam, the Netherlands; f Department of Internal Medicine, Section Geriatrics, Amsterdam UMC, Vrije Universiteit Amsterdam, the Netherlands; g Amsterdam Neuroscience, Neurodegeneration, Amsterdam, the Netherlands; h Department of Neurology, UMC Utrecht Brain Center, University Medical Center Utrecht, Utrecht, the Netherlands

**Keywords:** Carotid occlusive disease, Cerebrovascular disease, Heart failure, Nitrogen dioxide, Vascular cognitive impairment

## Abstract

**Background:**

We investigated if ambient air pollution and neighbourhood walkability associate with presence and progression of cerebral small vessel disease (CSVD) in patients with cardiovascular disorders along the heart-brain axis.

**Methods:**

Data from patients with heart failure, carotid occlusive disease, or vascular cognitive impairment (30% women; mean age 69.0 years) of the Heart-Brain Connection study were used. Brain parenchymal fraction (BPF), white matter hyperintensity fraction (WMH), cerebral blood flow (CBF), and incident vascular lesions were assessed with 3T brain MRI at baseline (n = 300) and at two years follow-up (n = 181). Incident clinical cerebrovascular events were assessed with questionnaires and telephone interviews up to five years. Annual residential nitrogen dioxide (NO_2_; µg/m^3^) concentration was linked to participants at baseline. Neighbourhood walkability was measured using the Dutch walkability index within a 500-m Euclidean buffer around each residential-area centroid (range 0–100). Associations were analyzed using age- and sex-adjusted logistic regression and repeated-measures linear mixed models.

**Results:**

At baseline, no association was found between NO_2_ or walkability and BPF, WMH, CBF, infarcts or microbleeds. Walkability (per 10 units) was associated with lower odds for having infarcts at baseline (OR 0.82, 95%CI 0.96–0.99) and attenuated age-related WMH increases by 0.01 SD (95%CI 0.00–0.02) per year. In longitudinal analyses, no associations between NO_2_ or walkability and progression of vascular brain lesions, cerebrovascular events, or mortality were found.

**Conclusion:**

Although air pollution and neighbourhood walkability were not strongly associated with CSVD progression, stroke, or mortality, higher walkability appeared to modestly attenuate age-related increases in WMH.

## Highlights

• In cardiovascular patients, NO₂ and walkability were studied related to CSVD.
• NO₂ and walkability showed no association with most CSVD MRI markers.
• Higher walkability was associated with fewer brain infarcts at baseline.
• Higher walkability modestly slowed age-related WMH progression.
• No association with CSVD progression, stroke, or mortality during follow-up.

## 1 Introduction

Cerebrovascular disease is the second leading cause of death and the third leading cause of loss in productive years worldwide [1]. Cerebral small vessel disease (CSVD) is a common manifestation of vascular disease and a frequent comorbidity in individuals with cardiovascular disease, characterized by diverse features on brain imaging or pathological examination. It is thought to have resulted from disease of the small blood vessels that perforate into the white matter, deep brain structures, and cortical gray matter [2]. CSVD is considered to cause 25% of ischemic strokes and most haemorrhagic strokes in older patients [3]. It is the most common cause of age-related disabilities such as vascular cognitive impairment, vascular and mixed dementias (contributing to about 50% of dementias), gait and balance problems, and mood disorders in ageing people [3]. Despite being common, the underlying pathogenesis of CSVD is not fully understood [4]. Previous studies have highlighted the need for elucidation of the role of environmental exposures to understand variation in CSVD development and clinical symptoms [4].

Ambient air pollution is suggested to be a contributor in cerebrovascular disease, especially in strokes [[5], [6], [7]]. There is some evidence showing that ambient air pollution would also be linked to CSVD [5]; however, the findings are somewhat inconsistent. Further, neighbourhood walkability, defined as the extent to which neighbourhoods are conducive to walking, has been linked to a lower risk of cerebrovascular disease [8,9]. The literature is limited on the association between walkability and CSVD.

Generally, the cardiovascular effects of environmental exposures are small among healthy individuals. For example, the worldwide burden of stroke attributed to air pollution has been estimated to be around 1.2–3.2% [10]. However, in high-income countries, 8.8% of stroke-related disability-adjusted life years are due to ambient air pollution [11]. Little evidence is available about the impact of air pollution or walkability in people with existing cerebrovascular or cardiovascular disease. Most previous evidence about the impact of environmental exposures has focused on the risk of developing cerebrovascular disease, rather than on how the exposures influence disease recurrence or progression in affected individuals. Vascularly comprised patients might be more susceptible for environmental factors in their residential environment [10]. For example, air pollution can exacerbate inflammation and oxidative stress, which further strain the cardiovascular system[12]. In vulnerable populations, environmental exposures may have a magnified impact [13,14]; and they can potentially worsen existing conditions and disease progression [15].

Recently, a Heart-Brain Connection Study (HBC) paper was published showing no association between air pollution and cognition in patients with cardiovascular disorders along the heart-brain axis [16]. Another recent HBC paper showed that neighbourhood walkability was positively associated with persistence of cognition [17]. It remains unknown if air pollution and neighbourhood walkability associate with presence or progression of CSVD. Furthermore, it remains unknown if air pollution or neighbourhood walkability are associated with risk of future cerebrovascular events.

In this study, we examine cross-sectional and longitudinal associations of objectively measured air pollution and neighbourhood walkability with measures of CSVD in the same HBC cohort, i.e. in patients with cardiovascular disorders along the heart-brain axis (i.e., heart failure (HF), carotid occlusive disease (COD), and vascular cognitive impairment (VCI)). We consider brain parenchymal fraction (BPF), white matter hyperintensity volume and cerebral blood flow (CBF) as measures of CSVD, as well as incident vascular lesions (i.e., cortical infarcts, subcortical infarcts, lacunes, cerebellar infarcts, microbleeds and enlarged perivascular spaces). We also study the role of air pollution and neighbourhood walkability in the risk of having future cerebrovascular events and of mortality. It is hypothesized that individuals living in neighbourhoods with higher levels of air pollution or lower neighbourhood walkability at baseline have more CSVD findings at baseline as well as increased progression during the follow-up.

## 2 Methods

### 2.1 Study design and study sample

The HBC study investigates the relationships between (cardio)vascular factors, the hemodynamic status of the heart and the brain, cerebrovascular disease, and cognitive functioning using data from patients with cardiovascular disorders along the heart-brain axis (i.e., HF, COD and VCI) [18]. Data were collected from cardiology, memory, and neurology outpatient clinics from four sites in the Netherlands: Amsterdam UMC, location VU University medical center (AUMC-VUmc) in Amsterdam (151 participants), Leiden University Medical Center (LUMC) in Leiden (98 participants), Maastricht University Medical Center (MUMC) in Maastricht (155 participants), and University Medical Center Utrecht (UMCU) in Utrecht (162 participants).

The baseline data collection took place from 2014 to 2017. The two-year follow-up measurement was conducted from 2016 to 2019. The study protocol with detailed inclusion and exclusion criteria per participant group has been described in the study by Hooghiemstra et al. [18]. In short, inclusion criteria for all participants were age ≥50 years, ability to undergo cognitive testing, and independence in daily life. Excluded were those with a contraindication for magnetic resonance imaging (MRI), life-threatening disease with a life expectancy of <3 years, neurodegenerative disease other than VCI or Alzheimer disease, neurologic or psychiatric diagnosis that could affect cognitive performance, atrial fibrillation upon enrolment, and premature ventricular contractions of >10% of heartbeats.

HF was defined following European Society of Cardiology 2012 Guidelines, and patients were clinically stable for at least six months before enrolment [18]. COD was defined as significant stenosis >80% or occlusion of an internal carotid artery not scheduled for surgical intervention. VCI was defined as cognitive complaints in the absence of dementia, with at least moderate white matter lesion (Fazekas>1) and/or (lacunar) infarct(s) and/or intracerebral (micro-)hemorrhage(s) on brain MRI or mild white matter lesions (Fazekas=1) in the presence of vascular risk factors or a history of manifest vascular disease, with a Mini-Mental State Examination score of ≥20.

In total there were 566 participants at baseline (Fig. 1). Of them, 162 had HF, 109 had COD, 166 had VCI and 129 were reference participants. The latter group was excluded from the current sample as we aimed to investigate the impact of environmental exposures among persons with an existing cardiovascular disorder. Of the remaining sample of 437 patients, information on six-digit postal codes at baseline was lacking for all patients who were included at the MUMC in Maastricht (n = 129). Therefore, these patients were excluded from the current study. In addition, we excluded eight participants who relocated between baseline and follow-up. The final baseline sample of the present study was 300 (Table 1). Those excluded due to missing postal code data or due to relocation during the follow-up had lower mean age (67.6 (SD 9.4) vs 69.0 (SD 8.8)), and higher proportion of them were females (38.0%vs 29.7%), than in the final baseline sample. Of the excluded, 47.4% had HF, 7.3% had COD, and 45.3% had VCI compared to 32.3%, 33.0% and 34.7% in the final baseline sample, respectively.

**Fig. 1:**
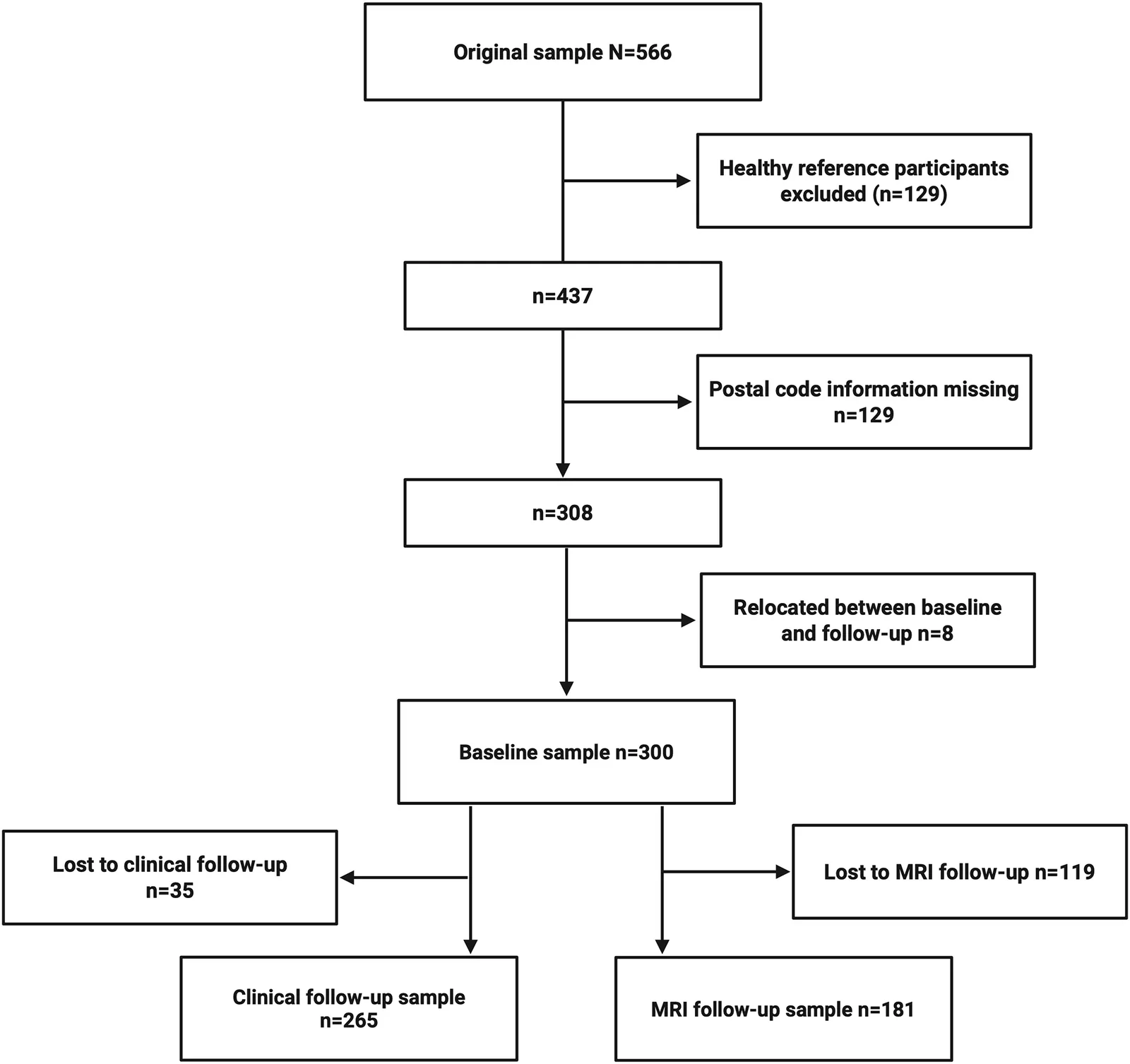
Flow-chart of the study inclusion and follow-up samples.

**Table 1:** Patient characteristics at baseline and at two years brain MRI follow-up.

	Baseline sample (N = 300)	MRI follow-up sample (N = 181)
	mean (SD) / n (%)	mean (SD) / n (%)
Baseline age in years	69.0 (8.8)	67.6 (8.4)
Female sex	89 (29.7%)	58 (32.0%)
Patient group		
HF	97 (32.3%)	49 (27.1%)
COD	99 (33.0%)	67 (37.0%)
VCI	104 (34.7%)	65 (35.9%)
Site		
Amsterdam	128 (42.7%)	83 (45.9%)
Leiden	45 (15.0%)	23 (12.7%)
Utrecht	127 (42.3%)	75 (41.4%)
Educational level		
Low	76 (25.3%)	49 (27.1%)
Intermediate	99 (33.0%)	62 (34.3%)
High	123 (41.0%)	70 (38.7%)
Area-level SES	0.34 (0.99)	0.38 (0.97)
Smoking		
Never	55 (18.3%)	34 (18.8%)
Former	187 (62.3%)	116 (64.1%)
Current	56 (18.7%)	31 (17.1%)
Hypertension	191 (63.7%)	114 (63.0%)
Hypercholesterolemia	190 (63.3%)	124 (68.5%)
Diabetes	60 (20.0%)	31 (17.1%)
Previous stroke		
Ischemic	79 (26.3%)	55 (30.4%)
Hemorrhagic	4 (1.3%)	2 (1.1%)
Undefined	3 (1.0%)	3 (1.7%)
Previous TIA	105 (35.0%)	68 (37.6%)
Family history of stroke	20 (6.7%)	12 (6.6%)
Family history of TIA	18 (6.0%)	11 (6.1%)
PM_2.5_	11.4 (1.2)	11.4 (1.3)
PM_10_	18.8 (1.5)	18.8 (1.5)
NO_2_	20.5 (4.4)	20.5 (4.3)
Walkability index	32.0 (15.1)	32.0 (14.8)

SD = standard deviation, HF = heart failure, COD = carotid occlusive disease, VCI = vascular cognitive impairment, SES = socioeconomic status, TIA = transient ischemic attack, PM_2.5_ = concentration of particulate matter with diameter <2.5 µm in µg/m^3^, PM_10_ = concentration of particulate matter with diameter <10 µm in µg/m^3^, NO_2_ = nitrogen dioxide in µg/m^3^. Information on history of stroke at baseline was missing for 2 (0.7%) participants and information on history of TIA at baseline for 3 (1.0%) participants.

The baseline sample of 300 participants was followed for radiological measures of CSVD for two years, and for cerebrovascular events or mortality for up to five years. Of the 300 baseline patients, 119 did not participate in the two-year follow-up brain MRI (Fig. 1). The remaining 181 form the final MRI follow-up sample of the current study (Table 1). Those who were lost to MRI follow-up were older at the baseline (mean age 71.2 (SD 8.9) vs 67.6 (SD 8.4)), and had lower proportion of females (26.1%vs 32.0%), than those who participated. Of those lost to follow-up, 40.3% had HF, 26.9% had COD, and 32.8% had VCI, compared to 27.1%, 37.0% and 35.9% in the MRI follow-up sample, respectively.

Of the same baseline sample of 300 participants, 35 (11.7%) were lost to follow-up for clinical cerebrovascular events and mortality, leaving the clinical five-year follow-up sample to be 265 patients (Fig. 1). Those lost to clinical follow up were older (mean baseline age 71.4 (SD 9.8) vs 68.7 (SD 8.6)), and had lower proportion of females (17.1%vs 31.3%), than the clinical follow-up sample. Of those lost to follow-up, 40.0% had HF, 31.4% had COD, and 28.6% had VCI, compared to 31.3%, 33.2% and 35.5% in the clinical follow-up sample, respectively.

### 2.2 Ethics statement

The HBC study was approved by the medical ethics committee of the Leiden University Medical Center, The Netherlands (P.14.002). Local medical ethics committees of the other sites approved the local implementation of the study. The study was conducted according to the Declaration of Helsinki. Written informed consent was obtained from all participants.

### 2.3 Exposures: air pollution and neighbourhood walkability

The HBC study was enriched with objectively measured environmental data on the six- and four-digit postal code-level. These data have been collected in the Geoscience and Health Cohort Consortium (GECCO) [19,20]. In all analyses, baseline information on air pollution and neighbourhood walkability was used.

We used concentration of nitrogen dioxide (NO_2_) as measure of air pollution. Based on a combination of model calculations and measurements from official measurement locations, the National Institute for Public Health and the Environment has estimated the spatial variation of annual average outdoor concentrations of air pollutants with a resolution of 25 × 25 m for various years in the Netherlands. In GECCO, these data have been processed and made available for six-digit postal code areas, i.e., administrative areas (average area size: 0.0025 km^2^) that include, on average, 15 households. Data on annual average outdoor concentration of NO_2_ in µg/m^3^ in six-digit postal code areas were linked to individuals who participated at baseline in the HBC, using their residential six-digit postal codes [20]. In the current study, NO_2_ data was studied per increase of 10 µg/m^3^.

Neighbourhood walkability within 500-m Euclidean buffer zones around the centroid of each participant’s residential six-digit postal code area was objectively measured using the Dutch neighbourhood walkability index. The walkability index has been developed in GECCO and is a composite measure combining six key spatial components that particularly facilitate walking. The components entail: population density, retail and service destination density, land use mix, street connectivity, green space density, and sidewalk density. The index ranged from 0 (low) to 100 (high) and was studied as per increase of 10. Detailed information about the construction of the neighbourhood walkability index, and its components, can be found elsewhere [17,21].

### 2.4 Brain MRI

Brain MRI imaging took place at baseline and at two-years follow-up. The standardized brain MRI protocol, including three‐dimensional T1‐weighted, T2 fluid‐attenuated inversion recovery (FLAIR) and susceptibility‐weighted imaging (SWI), has been described in detail elsewhere [18,22]. The MRI scans were acquired on Philips Ingenia 3T scanners at LUMC and UMCU and a Philips Gemini 3T PET-MR scanner at AUMC-VUmc (Philips, Best, the Netherlands).

The following brain MRI lesions were rated by the same experienced neuroradiologist at baseline and at two-years follow-up: presence of infarcts (i.e., cortical and non-lacunar subcortical infarcts; lacunar infarcts; cerebellar infarcts), cerebral microbleeds (lobar; non-lobar), and enlarged perivascular spaces. Brain infarcts, microbleeds, and perivascular spaces were rated according to the STandards for ReportIng Vascular changes on nEuroimaging (STRIVE) criteria [23,24]. Infarcts were subsequently manually segmented and masked out of the scans before further segmentation to increase the accuracy of the measured volumes [18,22].

For each participant, infarcts and other pathologies that potentially affect automatic tissue segmentation, were annotated by a neuroradiologist. Subsequently, the annotated infarcts and pathologies were manually segmented by trained students. In addition, a brain tissue and white matter hyperintensity segmentation method (Quantib B.V., Rotterdam, the Netherlands) was applied to T1-weighted and FLAIR MRI scans. Using the manual infarct segmentations, corrected volumes in mL of white matter hyperintensities, gray matter, normal appearing white matter, and infarcts were computed. Total brain volume in mL was calculated by summing these volumes. To correct for head size, BPF and white matter hyperintensity fraction (WMH) were calculated by dividing total brain volume and white matter hyperintensity volume by total intracranial volume (ICV) and expressing these as a percentage of ICV. Total ICV in mL was calculated by summing corrected volumes of white matter hyperintensities, gray matter, normal appearing white matter, cerebrospinal fluid, and infarcts including tissue loss. These corrected volumes were obtained using manual infarct segmentations. Other pathologies and incidental findings were not part of the total ICV. As an outcome, BPF represents the percentage of ICV that is occupied by brain tissue. Global brain atrophy is represented by smaller BPF. WMH represents the percentage of ICV that is occupied by white matter hyperintensities. Because of the highly skewed distribution of WMH, a natural logarithmic scale of it was used in analyses.

Whole brain partial volume corrected CBF in mL/100 g/min was examined with pseudo-continuous Arterial Spin Labelling (pCASL) (multi-slice two-dimensional Echo Planar Imaging (EPI) acquisition with background suppression; labelling duration = 1800 milliseconds; post-labelling delay = 1800 milliseconds; single shot EPI readout; resolution = 3 × 3 × 7 mm). The pCASL data were processed using the automated Iris pipeline, including a region-of-interest segmentation method and an ASL quantification method. Quantification of ASL data into CBF maps was based on a single-compartment model after the subtraction of labelled images from control images according to the recommended approach [25]. To scale the signal intensities of the subtracted ASL images to absolute CBF units, a separately acquired proton density weighted image (M0) was used. The quantification further included motion correction of the raw ASL data and partial volume correction. CBF was quantified in normal-appearing gray matter (NAGM) only. To obtain the NAGM mask for each participant, binary gray matter segmentation (Statistical Parametric Mapping, London, United Kingdom) was combined with the WMH and infarct mask. ASL-MRI analyses were performed in an independent core laboratory and was blinded to clinical data. Images with suboptimal quality (i.e., motion artefacts, incomplete ASL-sequence, or labelling errors) and images with dominant vascular artefacts and little tissue perfusion signal were not used for analyses.

### 2.5 Clinical cerebrovascular disease and mortality

The medical history of participants was recorded during a structured interview at baseline and incident conditions or events at two-year follow-up, as well as with telephone interviews at around five years of follow-up. Clinical presence of any cerebrovascular disease at baseline was defined as self-reported history of ischemic or hemorrhagic stroke, transient ischemic attack (TIA), or unspecified cerebrovascular event in the structured interview. In addition to history of any cerebrovascular disease, history of ischemic stroke or TIA were studied separately as baseline outcomes.

Any new cerebrovascular event, new stroke, new TIA, and mortality during the follow-up to five years were considered as the clinical outcomes in longitudinal analyses. In the two-years follow-up, clinical data on cerebrovascular disease incidence, type of cerebrovascular event, and admission to hospital or nursing home between the baseline and the follow-up visit were collected by history taking (n = 200). At the five-year follow-up, a similar structural interview was conducted on telephone with information on cerebrovascular disease incidence and type, and admission to hospital or nursing home (n = 138). Before the interview, participants were asked for permission to be contacted via telephone. In both follow-ups, for participants with cognitive problems or when the history of the participant was considered less reliable, with their permission, additional history was obtained from next of kin and/or the general practitioner. For deceased participants, the cause of death was obtained from Cause of Death Register of Statistics Netherlands and general practitioners.

### 2.6 Covariates

Covariates used in the main analyses included baseline age (in years) and sex as binary variable (females as reference category). In sensitivity analyses, models were further adjusted for patient group, site, educational level, smoking, hypertension, dyslipidemia, diabetes, and area-level socioeconomic status. For basic characteristics concentration of particulate matter with diameter <2.5 µm (PM_2.5_ in µg/m^3^) and concentration of particulate matter with diameter <10 µm (PM_10_ in µg/m^3^) were reported. Covariate data were collected at baseline by questionnaires, physical examination and a structured interview, except for area-level socioeconomic status which were obtained from national register data from the Netherlands Institute for Social Research [26], and for PM_2.5_ and PM_10_ which were obtained from GECCO.

### 2.7 Statistical analyses

Characteristics of the study samples and the area-level exposure measures are presented using descriptive statistics for the samples at baseline and at two-year follow-up. Linear and logistic regression analyses were conducted to examine the cross-sectional associations of NO_2_ or neighbourhood walkability with the continuous (i.e., BPF, WMH, CBF) and dichotomous measures (i.e., presence of lacunes, non-lacunar infarcts and microbleeds on brain MRI) of CSVD at baseline. Similarly, logistic regression was used to study the associations between NO_2_ or walkability and history of ischemic stroke or TIA at baseline.

Longitudinal change in BPF, WMH, and CBF was studied with repeated measures linear mixed models. We modeled longitudinal measurements of BPF, WMH, and CBF as dependent variables with NO_2_ or walkability as the exposures, age as the time scale, and adjusting for sex. The models included random intercept for each individual, but no random slope due to limited sample size. This means that the models accounted for individual variation in the starting levels of BPF, WMH, and CBF but not for individual variation in change of them over time. Adequacy of models was determined by examining the residuals for homoscedasticity and normality. The models were conducted as:

$$y_{ij}=\left(\beta_{0}+\upsilon_{0i}\right)+\beta_{1}\cdot age_{ij}+\;\beta_{2}\cdot exposure_{i}+\;\beta_{3}\cdot age_{ij}\cdot exposure_{i}+\beta_{4}\cdot sex_{i}\;+\;\varepsilon_{ij}$$

where $y_{ij}$ denotes the outcome measure (BPF, WMH, or CBF) for patient *i* at measurement *j*, β fixed effects, υ random effects (here random intercept), and ε residual error.

Also, logistic regression analyses were conducted to assess the longitudinal associations of NO_2_ and neighbourhood walkability at baseline with incident vascular lesions at two-year follow-up MRI and with new cerebrovascular events or mortality at follow-up.

Main analyses were adjusted for baseline age (when age was not used as time scale) and sex. Information on age and sex was complete for all participants. In sensitivity analyses, models were further adjusted for patient group, site, educational level, smoking, hypertension, dyslipidemia, diabetes, and area-level socioeconomic status. Analyses were performed in IBM SPSS Statistics (Version 29; IBM Corp, Armonk, New York, United States of America).

## 3 Results

### 3.1 Characteristics of sample

The baseline sample consisted of 300 patients (29.7% women; mean age 69.0 (SD 8.8) years). Of them, 97 (32.3%) had HF, 99 (33.0%) had COD, 104 (34.7%) had VCI (Table 1). A total of 181 patients (60.3% of the baseline sample) participated in the two-years follow-up brain MRI for radiological outcomes. Of them, 49 (27.1%) had HF, 67 (37.0%) had COD, and 65 (35.9%) had VCI. The mean follow-up time between the baseline brain MRI scan and the second brain MRI scan was 2.1 (SD 0.2) years, ranging from 1.8 to 3.1 years. Those participating in the follow-up MRI scan were slightly younger, more likely to be women, and lived in higher socioeconomic status areas compared to the baseline sample. The baseline air pollution exposures (NO_2_ mean 20.5 (SD 4.2) µg/m^3^) and walkability index (mean 32.0 (SD 15.1)) were similar.

Of the baseline sample of 300, a total of 265 (88.3%) patients were followed for clinical outcomes for up to five years. The mean follow-up from baseline until latest telephone interview for clinical outcomes was 5.4 (SD 0.8) years, ranging from 2.5 to 6.6 years.

### 3.2 Association of air pollution and neighbourhood walkability with BPF, WMH, CBF and vascular brain lesions at baseline and two-year follow-up

At baseline, no association was found between NO_2_ or walkability and BPF, WMH or CBF when adjusted for age and sex in main analyses (Table 2), or when adjusted for age, sex, patient group, site, educational level, smoking, hypertension, dyslipidemia, diabetes, and area-level socioeconomic status in sensitivity analyses (results not shown). In main linear mixed model analyses including age as time scale and sex as a covariate, no association between NO_2_ and level of BPF, WMH or CBF, or changes in them throughout age, were found (Table 3). Male sex was associated with 0.44 SDs (95% CI 0.17 to 0.71) decreased WMH, and with 0.50 SDs (95% CI 0.23 to 0.77) decreased CBF. Results of NO_2_ remained in sensitivity analyses (results not shown).

**Table 2:** Association between air pollution, neighbourhood walkability and cerebrovascular disease at baseline.

		NO_2_	Walkability index
	n (%) / mean (SD)	OR (95% CI) / B (95% CI)	OR (95% CI)
Any infarct	176 (66.7%)	0.58 (0.31–1.09)	0.82 (0.69–0.99)
Cortical infarcts	31 (14.8%)	0.50 (0.19–1.32)	0.81 (0.62–1.07))
Subcortical infarcts	6 (2.3%)	0.15 (0.02–1.15)	0.73 (0.40–1.33)
Lacunes	50 (26.2%)	0.82 (0.38–1.77)	0.83 (0.66–1.04)
Cerebellar infarcts	25 (10.7%)	1.73 (0.64–4.71)	1.02 (0.77–1.35)
Cerebral microbleeds	85 (29.0%)	0.95 (0.52–1.72)	0.99 (0.83–1.18)
Lobar	76 (25.9%)	0.97 (0.52–1.80)	0.98 (0.82–1.17)
Non-lobar	30 (10.2%)	0.82 (0.33–2.01)	1.13 (0.87–1.45)
Perivascular spaces	85 (31.1%)	0.57 (0.30–1.06)	0.92 (0.77–1.10)
BPF	0.77 (0.04)	0.51 (−0.35 to 1.37)	0.12 (−0.14 to 0.37)
WMH	0.01 (0.01)	0.11 (−0.26 to 0.48)	0.00 (−0.10 to 0.11)
CBF	50.90 (11.62)	2.74 (−0.38 to 5.86)	0.42 (−0.50 to 1.34)
Clinical CVA	86 (28.7%)	0.40 (0.05–3.38)	0.96 (0.81–1.15)
Ischemic CVA	79 (26.3%)	0.23 (0.03–2.06)	0.94 (0.78–1.13)
TIA	105 (35.0%)	0.42 (0.23–0.75)	0.73 (0.61–0.87)

N = 300. NO_2_ = concentration of nitrogen dioxide in µg/m^3^; OR = odds ratio; CI = confidence interval; ref = reference group; BPF = brain parenchymal fraction; WMH = white matter hyperintensities; CBF = cerebral blood flow; CVA = cerebrovascular accident (i.e., TIA, amaurosis fugax, or non-fatal ischemic stroke, ICH, or other stroke); TIA = transient ischemic attack. Perivascular spaces were considered present when they were rated as moderate or severe in the basal ganglia. Associations between NO_2_ or walkability and presence of lesions on brain MRI or presence of clinical cerebrovascular disease were analysed with logistic regression. Associations between NO_2_ or walkability and BPF, WMH, and CBF were analysed with linear regression. In regression analyses changes in NO_2_ concentration were studied per 10 ug and in walkability index per 10. Analyses were adjusted for baseline age and sex. Information on BPF and WMH were missing for 9 (3.0%) patients; information on CBF for 51 (17.0%) patients; information on any infarct or subcortical infarct for 36 (12.0%) patients; information on cortical infarcts for 90 (30.0%) patients; information on lacunes for 109 (36.3%) patients; information on cerebellar infarcts for 66 (22.0%) patients; information on cerebral microbleeds (both lobar and non-lobar) for 7 (2.3%) patients, information on perivascular spaces for 27 (9.0%) patients; information on any CVA and ischemic stroke for 2 patients (0.7%); and information on TIA for 3 patients (1.0%).

**Table 3:** Linear mixed models of progression of BPF, WMH, and CBF and NO_2_.

	Point estimate	95% CI
BPF		
Intercept	−0.10	−0.79 to 0.59
NO_2_	0.17	−0.14 to 0.48
Age	−0.05	−0.12 to 0.01
NO_2_*Age	−0.00	−0.04 to 0.03
Male sex	−0.18	−0.46 to 0.10
WMH		
Intercept	0.24	−0.43 to 0.91
NO_2_	0.00	−0.30 to 0.30
Age	0.06	−0.01 to 0.13
NO_2_*Age	0.00	−0.03 to 0.03
Male sex	−0.44	−0.71 to −0.17
CBF		
Intercept	−0.16	−0.82 to 0.51
NO_2_	0.25	−0.04 to 0.55
Age	−0.00	−0.08 to 0.07
NO_2_*Age	−0.01	−0.04 to 0.03
Male sex	−0.50	−0.77 to −0.23

Unstandardized point estimates show differences in outcome level (and change) per NO_2_ increase of 10 µg/m^3^ (per one year). BPF = brain parenchymal fraction; WMH = white matter hyperintensities; CBF = cerebral blood flow; NO_2_ = concentration of nitrogen dioxide in µg/m^3^; CI = confidence interval. Information on BPF and WMH were missing for 2 patients (1.1%); and on CBF for 51 patients (28.2%).

In main linear mixed model analyses investigating the relationship of walkability on outcomes, age was associated with increasing WMH (0.10 SDs (95% CI 0.07 to 0.14) per year) (Table 4). No association between walkability and WMH was observed; however, higher walkability (per 10 units) decreased the effect of age on WMH by 0.01 SDs (95% CI 0.00 to 0.02) per year. Male sex was associated with decreased WMH level by 0.47 SDs (95% CI 0.20 to 0.74). The associations of age, age*walkability interaction, and male sex remained in sensitivity analyses (results not shown). No association between walkability and BPF or CBF, or changes in them throughout age, were found in main analyses or in sensitivity analyses. Age was associated with decreasing BPF (−0.07 SDs (95% CI −0.10 to −0.03) per year) and male sex was associated with decreased level of CBF (−0.51 SDs; 95% CI −0.79 to −0.24) in main analyses.

**Table 4:** Linear mixed models of progression of BPF, WMH, and CBF and walkability.

	Point estimate	95% CI
BPF		
Intercept	0.16	−0.22 to 0.54
Walkability	0.03	−0.07 to 0.12
Age	−0.07	−0.10 to −0.03
Walkability*Age	0.00	−0.01 to 0.01
Male sex	−0.19	−0.46 to 0.09
WMH		
Intercept	0.22	−0.14 to 0.59
Walkability	0.02	−0.07 to 0.11
Age	0.10	0.07 to 0.14
Walkability*Age	−0.01	−0.02 to −0.00
Male sex	−0.47	−0.73 to −0.20
CBF		
Intercept	0.28	−0.10 to 0.66
Walkability	0.02	−0.06 to 0.11
Age	−0.03	−0.07 to 0.01
Walkability*Age	0.00	−0.01 to 0.01
Male sex	−0.51	−0.79 to −0.24

Unstandardized point estimates show differences in outcome level (and change) per walkability index increase of 10 (per one year). BPF = brain parenchymal fraction; WMH = white matter hyperintensities; CBF = cerebral blood flow; CI = confidence interval. Information on BPF and WMH were missing for 2 patients (1.1%); and on CBF for 51 patients (28.2%).

Considering other vascular lesions on brain MRI, 176 (66.7% of valid data) patients had visible infarcts of any kind at baseline (Table 2). Cerebral microbleeds were present in 85 patients (29.0%). Moderate or severely enlarged perivascular spaces in basal ganglia were seen in 85 patients (31.1%). Walkability was associated with lower odds for having infarcts (OR 0.82, 95% CI 0.96–0.99) at baseline when adjusted for age and sex (Table 2), but not after adjustment for age, sex, patient group, site, educational level, smoking, hypertension, dyslipidemia, diabetes, and area-level socioeconomic status in sensitivity analyses (results not shown).

On the two-years follow-up MRI, 30 patients (16.6% of the 181 rescanned) showed a new brain infarct (Table 5). New cerebral microbleeds were present in 20 patients (11.0%) and new moderate or severely enlarged perivascular spaces in basal ganglia were seen in 2 patients (1.1%). No associations between NO_2_ or walkability and new cerebrovascular lesions were found in main analyses (Table 5) or in sensitivity analyses (results not shown).

**Table 5:** Association between air pollution, neighbourhood walkability and progression of radiological CSVD during the two-year follow-up as new lesions.

		NO_2_	Walkability index
	n (%)	OR (95% CI)	OR (95% CI)
Any new infarct	30 (16.6%)	1.16 (0.43–3.14)	1.00 (0.76–1.31)
New cortical infarcts	9 (5.0%)	2.35 (0.42–13.27)	1.14 (0.72–1.79)
New subcortical infarcts	0 (0.0%)	n.a.	n.a.
New lacunar infarct	16 (8.8%)	0.82 (0.23–2.99)	0.97 (0.69–1.38)
New cerebellar infarcts	7 (3.9%)	1.08 (0.17–6.94)	0.88 (0.52–1.50)
New cerebral microbleeds	20 (11.0%)	1.11 (0.34–3.61)	1.04 (0.75–1.43)
Lobar	15 (8.3%)	1.01 (0.27–3.83)	0.98 (0.68–1.41)
Non-lobar	8 (4.4%)	1.01 (0.17–5.99)	1.09 (0.68–1.76)
New perivascular spaces	2 (1.1%)	n.a.	n.a.

N = 181. NO_2_ = concentration of nitrogen dioxide in µg/m^3^; OR = odds ratio; CI = confidence interval; n.a. = not applicable. Logistic regression analyses were adjusted for baseline age and sex. Information on any new infarct, new cortical infarct, new subcortical infarct, new lacunar infarct, and new cerebellar infarct were missing for 24 patients (13.3%); information on new cerebral microbleeds (both lobar and non-lobar) for 30 patients (16.6%); and information on new perivascular spaces for 1 patient (0.6%).

### 3.3 Associations of air pollution exposure and neighbourhood walkability with clinical cerebrovascular disease at baseline and during follow-up

Of the 300 patients at baseline, 79 (26.3%) had history of ischemic stroke, 4 (1.3%) of hemorrhagic stroke, 3 (1.0%) of undefined stroke, and 105 (35.0%) of TIA (Table 2). NO_2_ (OR 0.42, 95% CI 0.23–0.75) and walkability (OR 0.73, 95% CI 0.61–0.87) were associated with lower odds of having history of TIA when adjusted for age and sex in main analyses, but not after adjustment for age, sex, patient group, site, educational level, smoking, hypertension, dyslipidemia, diabetes, and area-level socioeconomic status in sensitivity analyses (results not shown).

During the follow-up from baseline up to five years, 44 (14.7%) patients died, 27 (9.0%) had any non-fatal cerebrovascular event (of whom two died later), and 35 (11.7%) were lost to follow-up. New ischemic stroke occurred in 3 (1.0%), and a new TIA in 19 (6.3%) patients. No associations between NO_2_ or walkability and new cerebrovascular event or mortality were found in main analyses (Table 6) or in sensitivity analyses (results not shown).

**Table 6:** Association of air pollution and neighbourhood walkability with mortality and incidence of new clinical cerebrovascular events during up to five years of follow-up.

		NO_2_	Walkability index
	n (%)	OR (95% CI)	OR (95% CI)
Mortality	44 (14.7%)	0.55 (0.25–1.22)	0.93 (0.75–1.16)
Any new CVA	27 (9.0%)	0.78 (0.30–2.02)	0.83 (0.62–1.10)
New ischemic stroke	3 (1.0%)	0.82 (0.06–11.24)	0.98 (0.42–2.27)
New TIA	19 (6.3%)	0.64 (0.21–2.00)	0.86 (0.62–1.20)

N = 265. NO_2_ = concentration of nitrogen dioxide in µg/m^3^; OR = odds ratio; CI = confidence interval; CVA = cerebrovascular accident (i.e., TIA, amaurosis fugax, or non-fatal ischemic stroke, ICH, or other stroke); TIA = transient ischemic attack, n.a. = not applicable. Logistic regression analyses were adjusted for baseline age and sex.

## 4 Discussion

In this sample of patients with cardiovascular disorders along the heart-brain axis, we could not demonstrate that air pollution or neighbourhood walkability were strongly associated with progression of CSVD or risk for stroke or mortality. Higher walkability might slightly protect from increasing WMH by age. Despite of the relatively small sample size, the findings may suggest that patients with cardiovascular disorders along the heart-brain axis are not particularly vulnerable to the harmful effects of air pollution and low neighbourhood walkability, at least not to a substantial extent.

Our hypothesis that air pollution and neighbourhood walkability could be associated with increased risk of cerebrovascular disease progression in these vulnerable groups was based on previous literature. Previous studies have linked NO_2_ (per 10 parts per billion) with 2% increased hospital admissions and overall mortality [15], although not with cardiovascular mortality [27], among patients with HF. The living environment is increasingly acknowledged to shape the disease prognosis of people with disorders along the heart-brain axis, although the associations are multifactorial. For example, among patients with carotid artery stenosis, living in areas with greater neighbourhood social disadvantage is associated with worse disease outcomes and higher risk for stroke and mortality [28]. Higher air pollution and lower neighbourhood walkability can be viewed as forms of environmental disadvantage. Here, we found that higher walkability might slightly protect from increasing WMH by age, which is in line with our hypothesis and previous literature.

It should be noted that, in our sample, almost all participants were exposed to air pollution levels higher than the recommended annual average exposure levels in the World Health Organization 2021 Global Air Quality Guidelines [29]. A potential explanation for the lack of associations in this population, where nearly everyone has relatively high exposure, could be the limited variation in air pollution exposure of NO_2_ [15]. However, other air pollution measures (i.e., PM_2.5_ and PM_10_) had even less variation, why we used NO_2_ as the single measure of air pollution exposure. It should be noted that NO₂ mainly reflects traffic emissions and may not capture the full burden of particulate pollution relevant to CSVD. The neighbourhood walkability index varied more among the patients in our study.

Our sample consisted of patients with cardiovascular disorders along the heart-brain axis, i.e., HF, COD, and VCI. Previously, air pollution has been linked to the increased risk of all these diseases [[30], [31], [32]]. Also, neighbourhood walkability has been linked to the decreased risk of many precursors of these diseases, such as hypertension [33], carotid artery atherosclerosis [34], and arterial stiffness [35], as well as of vascular dementia [36] and multimorbidity [37]. Here we studied if the two environmental exposures were associated with presence and progression of cerebrovascular disease in a cohort of persons with either HF, COD or VCI. However, it is possible that air pollution and neighbourhood walkability have different associations with cerebrovascular disease in these disease groups. We did not compare the disease groups or study them as potential association modifiers as the sample sizes per patient group were limited. Further, the associations might be different in sex- and age groups [38,39]. We encourage future studies to investigate the potential association modification based on patient characteristics.

Further, air pollution and neighbourhood walkability have been shown to have interactive associations with the risk of cerebrovascular disease in the general population [8]. Due to our small sample size, we did not investigate the interactive effects of the two exposures in this vulnerable population. Also, the association between neighbourhood walkability and cerebrovascular disease has been found to be partially explained by physical activity [40]. We decided not to conduct mediation analyses due to our limited sample size. Finally, the follow-up period was relatively short (i.e., two years for brain MRI and five years for cerebrovascular events and mortality) and there were no (substantial) changes in air pollution or neighbourhood walkability over time, and we did not examine whether changes in air pollution or neighbourhood walkability levels would be associated with cerebrovascular disease outcomes. We suggest these four considerations for future studies.

Other methodological limitations to address include that our baseline analyses might be biased with reversed causality if the persons with more severe cerebrovascular disease had moved to different areas due to their disease. This could be the case if, for example, persons living in less polluted rural areas moved to more polluted urban areas after having a stroke due to better healthcare facilities. To minimize this bias, we wanted to investigate the progression of cerebrovascular disease after baseline.

Also, due to limited sample size, we could not investigate individual variation in change of brain atrophy, WMH, or CBF as the linear mixed model structure did not allow it. For the same reason, we did not adjust the main analyses for other factors than age and sex. This limited adjustment may have resulted in residual confounding, particularly because neighbourhood walkability and air pollution may be associated with socioeconomic and lifestyle-related factors. We therefore conducted sensitivity analyses additionally adjusting for patient group, study site, educational level, smoking, hypertension, dyslipidemia, diabetes, and area-level socioeconomic status to assess the robustness of our findings to potential confounding by these factors. The findings remained largely unchanged, although the inclusion of multiple covariates further reduced statistical power and therefore warrants cautious interpretation. Also, exclusions and missing follow-up data may have introduced selection bias, potentially affecting the representativeness of the final sample. This should be considered when interpreting our findings.

A particular strength of this study is the integration of high-quality environmental exposure data on a detailed spatial scale together with repeated MRI-based data on brain health indicators in a multi-center study. The follow-up was up to five years for clinical outcomes, with only a 12% loss to follow-up. Around 60% of the sample participated in the brain MRI at two-year follow-up, allowing us to study both clinical and radiological progression cerebrovascular disease. Covariate information for main analyses was available for the total sample.

Overall, to our knowledge, this was the first study to investigate cross-sectional and longitudinal associations of air pollution and neighbourhood walkability with cerebrovascular disease in patients with cardiovascular disorders along the heart-brain axis. Our findings did not provide clear evidence that patients with disorders along the heart-brain axis are particularly vulnerable to the adverse effects of air pollution and low neighbourhood walkability. Higher walkability might slightly protect from increasing WMH by age. However, our findings should be interpreted cautiously given the limited exposure contrast, uniformly high air pollution levels, and limited statistical power for clinical endpoints. Although it can be challenging to gather repeated brain MRI data on large sample sizes of vulnerable patients, we suggest that future studies replicate our approach in a larger number of participants, over a longer follow-up period, and with more variation in the exposure levels. Also, future studies could not only consider exposure to air pollution in the residential environment but could use time-activity-weighted exposure measures that consider exposures at other significant places where individuals spend their time [15].

## Funding sources

This work is part of the Heart-Brain Connection crossroads (HBCx) consortium of the Dutch CardioVascular Alliance. HBCx has received funding from the Dutch Heart Foundation under grant agreements 2018–28 and CVON 2012–06.

The geo-data were collected as part of the Geoscience and Health Cohort Consortium, which was financially supported by the Netherlands Organization for Scientific Research (NWO) – the Netherlands Organization for Health Research and Development (project number: 91,118,017), and the Amsterdam UMC.

EJT is supported by EXPOSOME-NL. EXPOSOME-NL is funded through the Gravitation program of the Dutch Ministry of Education, Culture, and Science and the Netherlands Organization for Scientific Research (NWO grant number 024.004.017).

## Declaration of generative AI use

None used.

## Ethics approval and consent to participate

Ethical approval was obtained from the Review Board of Leiden University Medical Center (P.14.002). All participants have provided informed consent.

## Consent for publication

Not applicable.

## CRediT authorship contribution statement

**Ina Rissanen:** Writing – original draft, Methodology, Investigation, Formal analysis, Data curation, Conceptualization. **Erik J. Timmermans:** Writing – review & editing, Methodology, Investigation, Conceptualization. **Jeroen de Bresser:** Writing – review & editing, Investigation, Data curation. **Esther E. Bron:** Writing – review & editing, Investigation, Data curation. **Anna E. Leeuwis:** Writing – review & editing, Investigation, Data curation. **Majon Muller:** Writing – review & editing, Investigation, Data curation. **Matthias J.P. van Osch:** Writing – review & editing, Investigation, Data curation. **Betty M. Tijms:** Writing – review & editing, Investigation, Data curation. **Geert Jan Biessels:** Writing – review & editing, Supervision, Project administration, Methodology, Investigation, Funding acquisition, Data curation, Conceptualization. **Ilonca Vaartjes:** Writing – review & editing, Supervision, Resources, Project administration, Methodology, Investigation, Conceptualization.

## Declaration of competing interest

The authors declare the following financial interests/personal relationships which may be considered as potential competing interests:

Ina Rissanen reports receiving salary from Duodecim Publishing Company Ltd. Erik Timmermans reports financial support provided by Netherlands Organization for Scientific Research. Jeroen de Bresser reports financial support by Alzheimer Nederland (WE.03–2019–08 and WE.03–2024–13) and Medical Delta. Matthias van Osch is a member of the steering committee of a clinical trial by Alnylam Pharmaceuticals, his research group receives research support from Philips, and he receives funding from the Dutch Research Council and the Leducq Foundation. Geert Jan Biessels reports financial support was provided by Dutch Heart Foundation. If there are other authors, they declare that they have no known competing financial interests or personal relationships that could have appeared to influence the work reported in this paper.

## Data Availability

The data of the Heart Brain Study are available on request, provided that an agreement is made up with the steering group of the Heart-Brain Connection Consortium.
